# Microorganisms Associated with the Marine Sponge *Scopalina hapalia*: A Reservoir of Bioactive Molecules to Slow Down the Aging Process

**DOI:** 10.3390/microorganisms8091262

**Published:** 2020-08-20

**Authors:** Charifat Said Hassane, Mireille Fouillaud, Géraldine Le Goff, Aimilia D. Sklirou, Jean Bernard Boyer, Ioannis P. Trougakos, Moran Jerabek, Jérôme Bignon, Nicole J. de Voogd, Jamal Ouazzani, Anne Gauvin-Bialecki, Laurent Dufossé

**Affiliations:** 1 Laboratoire de Chimie et Biotechnologie des Produits Naturels, Faculté des Sciences et Technologies, Université de La Réunion, 15 Avenue René Cassin, CS 92003, 97744 Saint-Denis CEDEX 9, La Réunion, France; charifat.said-hassane@univ-reunion.fr (C.S.H.); mireille.fouillaud@univ-reunion.fr (M.F.); jean-bernard.boyer@univ-reunion.fr (J.B.B.); 2 Institut de Chimie des Substances Naturelles, CNRS UPR 2301, Université Paris-Saclay, 1, av. de la Terrasse, 91198 Gif-sur-Yvette, France; geraldine.legoff@cnrs.fr (G.L.G.); jerome.bignon@cnrs.fr (J.B.); Jamal.Ouazzani@cnrs.fr (J.O.); 3 Department of Cell Biology and Biophysics, Faculty of Biology, National and Kapodistrian University of Athens, 15784 Athens, Greece; asklirou@biol.uoa.gr (A.D.S.); itrougakos@biol.uoa.gr (I.P.T.); 4 Crelux GmbH, Am Klopferspitz 19a, 82152 Martinsried, Germany; Moran_Jerabek@wuxiapptec.com; 5 Naturalis Biodiversity Center, Darwinweg 2, 2333 CR Leiden, The Netherlands; nicole.devoogd@naturalis.nl; 6 Institute of Environmental Sciences, Leiden University, Einsteinweg 2, 2333 CC Leiden, The Netherlands

**Keywords:** *Scopalina hapalia*, Actinomycetes, *Bacillus*, Fungi, elastase inhibition, tyrosinase inhibition, CDK7 inhibition, Fyn kinase inhibition, catalase activation, sirtuin 1 activation

## Abstract

Aging research aims at developing interventions that delay normal aging processes and some related pathologies. Recently, many compounds and extracts from natural products have been shown to delay aging and/or extend lifespan. Marine sponges and their associated microorganisms have been found to produce a wide variety of bioactive secondary metabolites; however, those from the Southwest of the Indian Ocean are much less studied, especially regarding anti-aging activities. In this study, the microbial diversity of the marine sponge *Scopalina hapalia* was investigated by metagenomic analysis. Twenty-six bacterial and two archaeal phyla were recovered from the sponge, of which the *Proteobacteria* phylum was the most abundant. In addition, thirty isolates from *S. hapalia* were selected and cultivated for identification and secondary metabolites production. The selected isolates were affiliated to the genera *Bacillus*, *Micromonospora*, *Rhodoccocus*, *Salinispora*, *Aspergillus*, *Chaetomium*, *Nigrospora* and unidentified genera related to the family *Thermoactinomycetaceae*. Crude extracts from selected microbial cultures were found to be active against seven targets i.e., elastase, tyrosinase, catalase, sirtuin 1, Cyclin-dependent kinase 7 (CDK7), Fyn kinase and proteasome. These results highlight the potential of microorganisms associated with a marine sponge from Mayotte to produce anti-aging compounds. Future work will focus on the isolation and the characterization of bioactive molecules.

## 1. Introduction

As the world’s global population ages, an increase in the prevalence of a variety of age-related diseases such as inflammatory disorders, neurodegenerative and cardiovascular diseases, as well as cancer, is observed. Even though the use of the term “cause” can be debated, aging was identified as the main risk factor for many age-related diseases [1,2]. Over recent years, research about aging effects has experienced unprecedented advances, particularly with the discovery that delaying of aging (with genetic, dietary and pharmacological approaches) can impede the onset or progress of age-related diseases [3,4,5,6]. These interventions aim to slow down the effect(s) of normal aging, prevent age-related diseases and increase quality of life. In this context, we selected a set of biological targets relevant for our investigation on naturally occurring anti-aging agents. Elastase is a member of the chymotrypsin family of proteases and is primarily responsible for the degradation of elastin, which is an important protein giving elasticity to arteries, lungs and skin. Elastin breakdown through the action of elastase results in visible skin changes like wrinkles [7,8,9]. Tyrosinase is a major enzyme in melanin biosynthesis which determines the color of hair and skin. Increased tyrosinase activity was observed in hyperpigmentation disorders; conversely downregulation of tyrosinase activity led to melanogenesis inhibition [10,11]. Therefore, inhibition of elastase and tyrosinase is one of the most prominent strategies used to fight skin aging. Among the considered mechanisms of aging, the oxidative stress plays a substantial role [12]. As increased levels of reactive oxygen species (ROS) contribute to the pathogenesis of numerous diseases including age-related disorders, identifying compounds which have the capacity to increase the intracellular antioxidant defense can be useful to prevent these disorders [13]. Catalase is one of these antioxidant enzymes and prevents cell oxidative damage (i.e., the formation of oxidized proteins, lipids and/or DNA) by converting hydrogen peroxide (H_2_O_2_), which is continuously produced by metabolic reactions, to water (H_2_O) and oxygen (O_2_) [14]. Sirtuin 1 (Sirt1) belongs to the conserved sirtuin family (Sirt 1–7) of nicotinamide adenine dinucleotide (NAD^+^) dependent protein deacetylases [15]. Identified as key regulators of caloric restriction (CR), these proteins represent one of the most promising targets for anti-aging approaches [16,17]. Indeed, CR is so far the only effective way known to extend the lifespan and healthspan of a number of organisms without genetic or pharmacological intervention [18,19]. Studies have shown that upregulation of these proteins alleviates the symptoms of aging as well as of age-related diseases and induces physiological changes that are similar to CR [20,21]. CDK7 belongs to the Cyclin-dependent kinases (CDKs), best known for their critical roles in cell cycle regulation. However, this protein is also involved in other physiological process like DNA repair and transcription [22,23,24]. Recent reports demonstrated that CDK7 is crucial for the pathogenesis of certain cancer types driven by RNA polymerase-II-based transcription like triple-negative breast cancer, high-grade glioma or peripheral T-cell lymphomas [25,26,27,28]. The proteasome is a multi-subunit enzyme that is essential in cell function as it plays a central role in the regulation of protein homeostasis [29,30]. Cancer cells rely on the proteasome activity to maintain the protein homeostasis required for their enhanced metabolism and unrestricted proliferation. Therefore, inhibition of proteasome function emerged as a powerful strategy for anti-cancer therapy, especially in haematological malignancies [31]. Fyn is a non-receptor tyrosine kinase belonging to the Src family kinase, which is an important class of molecules in human biology. Recent studies highlight its involvement in signaling pathways leading to the pathogenesis of Alzheimer’s disease [32,33]. It has been demonstrated that Fyn interacts with both protein Tau and amyloid β-peptide, two key players responsible for the major pathologic hallmarks of Alzheimer’s disease [34,35]. These lines of evidence identified Fyn kinase inhibition as a novel approach therapy of particular interest.

Amongst the compounds with anti-aging activities discovered recently, many are natural products, some of which are drugs used in the clinic [36,37]. For example, numerous natural products or extracts from plants and microorganisms are widely used as cosmetic or cosmeceutical ingredients because of their antioxidant, anti-elastase and anti-tyrosinase activities [38,39,40,41,42]. However, some of natural and synthetic anti-tyrosinase compounds used as hypopigmenting agents may induce side effects following chronic exposure [43]. It is also worth noting that the first potent sirtuin activating compounds (STACs) included plant-derived metabolites such as resveratrol [21]. Since then, molecules, structurally related to resveratrol or not, have been developed. Almost all sirtuin activators described to date target Sirt1. Furthermore, marine natural products have been shown to be an interesting source of kinase as well as proteasome inhibitors for cancer and neurodegenerative diseases [44,45,46,47]. Salinosporamide A, isolated from marine actinomycete *Salinispora tropica*, is one of the most potent proteasome inhibitors and is currently undergoing clinical trials for cancers [48]. So in order to widen the chemical diversity of bioactive compounds, exploration of understudied biological resources or geographical areas is gaining importance.

In our search for natural products with bioactivities against our set of selected biological targets, we investigated the marine sponges from Mayotte, a French island located in the Comoros archipelago, recognized as a hotspot of biodiversity. This island hosts a variety of marine tropical ecosystems which are of major ecological value and are yet poorly studied. During this investigation, a marine sponge *Scopalina hapalia* exhibited interesting anti-aging activities showing moderate elastase inhibition and high Fyn kinase inhibition. The few reported studies investigating sponges from this geographical area did not consider, thus far, their associated microorganisms and their biotechnological potential [49,50,51,52,53,54,55,56]. The present study aims at investigating the potential of microorganisms isolated from *S. hapalia* to produce secondary metabolites with anti-aging activity. First, the prokaryotic diversity associated with *S. hapalia* was characterized by targeted 16S rRNA sequencing. Then, a cultivation approach was carried out to isolate microorganisms from *S. hapalia* with a special attention given to actinomycetes and filamentous fungi. Members of the *Bacillales* order were also identified and included in this study because of well-known representative producers of important secondary metabolites [57,58]. Finally, their potential to produce bioactive compounds with anti-aging activities was assessed by screenings for elastase, tyrosinase, CDK7, proteasome and Fyn kinase inhibitory activity, as well as for catalase catalase or sirtuin 1 activation.

## 2. Results and Discussion

### 2.1. Composition of the Prokaryotic Community of Scopalina hapalia

To explore the diversity of the microbial community associated with *Scopalina hapalia*, a culture-independent approach was implemented using targeted 16S rRNA and ITS2 metagenomics.

After quality-filtering, 16 353, 47 630, 37 213 and 46 370 sequences, respective to the V1–V3, V3–V4, V4–V5 regions of the 16S rRNA gene and to the ITS2 region, were obtained from the genomic DNA extracted from *Scopalina hapalia*. These sequences respectively clustered into 337, 408, 355 and 32 Operational Taxonomic Units (OTUs) at 97% similarity from the sequencing of V1–V3, V3–V4, V4–V5 and ITS2 regions (Table 1). Rarefaction curves were constructed and indicated a good recovery of OTUs from the sequencing depth (Figures S1–S3). The OTU richness and diversity estimations were obtained from rarefied data sets (Table 1). The alpha diversity analysis showed that the observed richness (observed OTUs) is slightly equivalent to the estimated richness (Chao1), suggesting that rare OTUs represent a small portion of the sponge microbial community (Table 1). The Shannon index from the three regions sequenced seems to indicate that *Scopalina hapalia* has a diversified microbial community.

Unfortunately, the selected primer pair used to amplify fungal ITS2 (Internal Transcribed Spacer 2) region from the total genomic DNA of *S. hapalia* did not yield satisfactory results. Among the 46,370 (100%) quality-filtered reads, 43,669 (94.18%) reads were unclassified and 573 (1.24%) belonged to Metazoa. In total, 2128 (4.59%) reads of fungi were obtained and classified into three different phyla: Ascomycota (4.29%—8 different OTUs), Basidiomycota (0.15%—7 different OTUs) and other (0.15%—7 different OTUs). Two OTUs belonged to the classes Sordariomycetes and Saccharomycetes (Ascomycota), while five OTUs belonged to the classes Agaricomycetes and Malasseziomycetes (Basidiomycota). Fifteen OTUs could not be classified either at the phylum or class level. Ascomycota and Basidiomycota divisions are widely distributed in marine sponges with Ascomycota being the dominant one [59]. Members of Sordariomycetes, Malasseziomycetes and Agaricomycetes were previously reported from specimens of *Scopalina* collected in Australia [59]. The very low number of fungal reads in comparison to the number of non-targeted DNA is in line with previous studies examining fungal communities in marine sponges by cultivation-independent methods [60,61,62]. One possible explanation for this result could be the lower abundance of fungi than that of bacteria in marine sponges. As a typical milliliter of seawater contains 10^3^ fungal cells and 10^6^ bacteria, without specific filtration from the sponge, the fungal cells could be much lower [63].

From the entire data sets, 26 bacterial and two archaeal phyla were identified (Figure S4). This is a high level of phyla diversity for a Low Microbial Abundance (LMA) sponge. Indeed, sponges belonging to the genera *Scopalina* (order Scopalinida) have been characterized as LMA sponges, which are generally assumed to have a less diverse bacterial communities (low phylum-level diversity) than high microbial abundance (HMA) sponges [64,65]. Nonetheless, our result is in agreement with the study by de Voogd et al. (2018), which reported that some LMA sponges from Mayotte harbored a more diverse bacterial community at the phylum level than HMA sponges (e.g., *Stylissa carteri*, order Scopalinida: 29) [66]. Our result supports their conclusion that a true dichotomy between HMA and LMA sponges does not appear to exist [66]. In our study, the *Scopalina hapalia* community was dominated by *Proteobacteria* (62.58%, 94.03% and 57.21% relative abundance in V1–V3, V3–V4 and V4–V5 data sets), *Cyanobacteria* (16.77%, 0.05% and 11.08%), *Planctomycetes* (11.71%, 0.06% and 9.90%), *Bacteroidetes* (4.77%, 1.24% and 17.99%) and *Actinobacteria* (1.68%, 2.82% and 1.30%). From the other 23 phyla, each contributed <1.16% to the *S. hapalia* data set sequences. On the genus and family level, the five most abundant taxa were an unidentified genus of the *Endozoicimonaceae* family (*Gammaproteobacteria*) (6.46%, 48.03%, 6.36%), a member of the uncultured order EC94 (*Betaproteobacteria*) (9.88%, 11.59%, 10.37%), *Ruegeria* (*Alphaproteobacteria*) (5.82%, 5.87%, 9.98%), *Labrenzia* (*Alphaproteobacteria*) (7.37%, 5.60%, 8.41%) and *Aquimarina* (*Flavobacteriia*–*Bacteroidetes*) (3.72%, 0.05%, 14.29%). Most of the sequences from these taxa corresponded to highly abundant OTUs.

Previous studies based on 16S rRNA gene sequencing or metagenomic analysis revealed that the prokaryotic microbiome of *Scopalina* is dominated by Proteobacteria (*Gammaproteobacteria* and *Betaproteobacteria*) and *Cyanobacteria* [65,67,68,69]. Even though the diversity of *Scopalina* symbionts has been addressed in some studies, it is difficult to do comparative work due to methodological, sample processing and data analysis differences. However, our results are consistent with previous results showing that *Scopalina* sponges are dominated by the LMA-indicator phylum *Proteobacteria* [65], and further support the notion that the community of LMA sponges is dominated by the classes *Alpha*-, *Beta*- and *Gammaproteobacteria* and *Flavobacteriia* [70]. 

### 2.2. Isolation and Identification of Microorganisms Associated with Scopalina hapalia

A number of studies investigating microbial communities of *S. ruetzleri* and *Scopalina* sp. have shown that these sponges host phyla (Actinobacteria, Cyanobacteria, Proteobacteria) with potential for natural products synthesis [67,68,71,72,73]. As the crude extract of *S. hapalia* exhibited interesting biological activities, isolation studies focusing on actinomycetes and filamentous fungi were carried out. Homogenates from *S. hapalia* were plated on a range of eight isolation media including selective ones for actinomycetes and fungi. In total, 124 microbial strains were isolated, among which 10 putative actinomycetes and three filamentous fungi were recovered. R2A media exhibited the highest recovery of isolates (31) and SFM recovered only three isolates. As for the selected media for the isolation of actinobacteria, MBA exhibited the highest recovery (4) followed by SCAM (3), R2A (2) and A1BFe+c (1). In terms of diversity, MBA and SCAM gave isolates belonging to three different species, followed by R2A (2) and A1BFe+c (1). Thirty microbial strains, including the three filamentous fungi, were selected for identification.

#### 2.2.1. Identification of Culturable Actinomycetes Associated with Scopalina hapalia

A comparison of the partial 16S RNA gene sequences of the 10 putative actinomycetes strains against EzBioCloud 16S database exhibited 99–100% sequence similarities with validly described species (Table S1). The analysis revealed the phylogenetic affiliations to three different genera (*Micromonospora* (six strains), *Salinispora* (three strains) and *Rhodococcus* (one strain)) representing two families: Micromonosporaceae and Nocardiaceae.

*Micromonospora* and *Rhodococcus* have been reported to be amongst the dominant actinobacterial genera in marine environment along with *Streptomyces* [74]. *Salinispora* is an obligate marine actinomycete originally discovered in sediments, then found in sponges [75,76]. It is noteworthy to indicate the presence of the species *Micromonospora endophytica*, formerly known as *Jishengella endophytica* and cultivated from *Acanthus illicifolius* root collected from a mangrove zone in China [77,78]. To the best of our knowledge, this is the first report of the genus *Salinispora* in this geographical area and also the first one about the isolation of *M. endophytica* from any marine invertebrate, especially from sponges. As the Mayotte lagoon includes a mangrove, this result seems to make sense. Representatives of the genera *Micromonospora*, *Rhodococcus* and *Salinispora* have previously been isolated from the specimen *Scopalina ruetzleri* from the Caribbean [71], suggesting that these genera might be part of the specific symbionts associated to the genus *Scopalina*.

#### 2.2.2. Identification of Bacillales Strains Isolated from Scopalina hapalia

The remaining 17 strains shared >98% sequence identity with validly described species within the order Bacillales (Table S2). Fifteen strains were closely related to the genus *Bacillus* and one strain was closely related to previously cultured bacteria DQ448769, for which the nearest type strain is *Laceyella sacchari* belonging to the Thermoactinomycetaceae family [79]. One strain (SH-39) shared <98% sequence identity with DQ448769 suggesting that this might represent a new phylotype. According to Kim and co-workers, a threshold of 98.65% 16S rRNA gene sequence similarity can be considerate to differentiate two species [80]. Most of the *Bacillus* isolates were affiliated either to *B. licheniformis* (AE017333) (7/15) or to *B. paralicheniformis* (KY694465) (7/15). Members of the *Bacillus* group, including *B. licheniformis*, are common inhabitants of marine environments and have been often isolated from sponges and other organisms [79,81]. *B. paralicheniformis,* closely related to *B. licheniformis* and also retrieve in sponges, was described in 2015 from soybean-based fermented paste [82,83]. Representative members of *Bacillus* were previously reported from *Scopalina* sp. [68]. 

It is worth noting that even though 16S rRNA gene sequencing has proved to be a powerful tool for bacterial identification, it tends to present limitations in distinguishing between closely related species like *Bacillus*, *Micromonospora* and *Salinispora* [84]. This is especially true for the genus *Salinispora,* as six novel species sharing >99% 16S RNA sequence similarity with the three currently recognized species were recently described [85]. Still, this method was chosen because it is a rapid and easy approach for the reliable identification of unknown strains.

#### 2.2.3. Identification of Filamentous Fungi

From the culture-dependent approach, only three fungal strains were recovered. This low number of fungal isolates is consistent with a previous study on the cultivable fungi from Panama, where three fungal strains were recovered from three different specimens of *Scopalina* [86]. On the other hand, more than 19 fungal isolates were recovered from three replicates of *Scopalina* sp. collected in Australia [59]. Our results on the fungal community housed in this specimen of *Scopalina hapalia* are in line with previous works demonstrating that: (1) the number of fungal strains isolated per sponge could vary between locations [87] and (2) discrepancies can be obtained between the molecular and culture approach [59].

The molecular identification revealed a phylogenetic affiliation to the three different genera: *Aspergillus* (class Eurotiomycetes, order Eurotiales), *Chaetomium* (class Sordariomycetes, order Sordariales) and *Nigrospora* (class Sordariomycetes, order Trichosphaeriales) (Table S3). When compared to the ITS2-amplicon sequencing, which affords 22 fungal OTUs, it seems that further efforts would be needed to reveal the entire culturable diversity of fungi present in this sponge microbiome.

Previous works showed that many of the fungal strains isolated from sponges could be classified within the order Eurotiales as it included ubiquitous genera: *Aspergillus* and *Penicillium,* which are prolific sporulating and non-fastidious fungi, and the order Hypocreales (*Acremonium* and *Trichoderma*) [73,74,75,76,77]. Representative of the genus *Penicillium* (Eurotiales), *Acremonium* and *Trichoderma* (Hypocreales) were cultured from *Scopalina* sp. during previous studies [59,86]. The genera *Chaetomium* and *Nigrospora* were previously reported from studies of sponge-derived fungi [87,88,89], though to the best of our knowledge this is the first report of this newly described species *N. aurantiaca* from sponge [90].

### 2.3. Biological Assays

Thirty of the 124 isolates were selected for the production of biologically active metabolites. These isolates belong to genera known to produce novel bioactive metabolites or to genera/species that have not been extensively studied (Tables S1–S3). These 30 isolates were cultivated in liquid (LM) and solid (SM) Marine Broth media. Liquid- and solid-state fermentations were coupled with in situ solid-phase extraction using XAD-16 resin. After five and fifteen days of cultivation, the resin and biomass were extracted successively with ethyl acetate (EA) and methanol (MeOH) solvents. Overall, 118 microbial crude extracts were obtained and evaluated for their bioactivity(-ies) against seven different molecular targets.

#### 2.3.1. Anti-Elastase Activities

**Figure 1:**
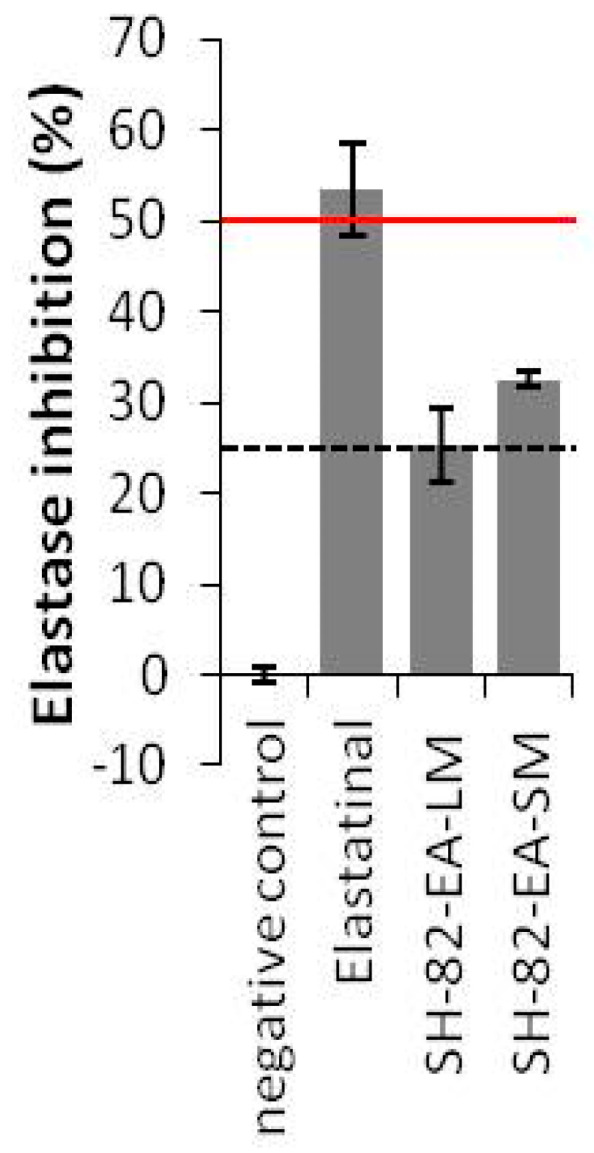
Percentage inhibition of elastase activity by selected microbial crude extracts. Only the most active crude extracts are shown. The code per isolates is outlined in Table S1. Bars, ±SD; *n* ≥ 2.

Preliminary screening on purified elastase identified three extracts with moderate activities (25–50% inhibition) as compared to a known elastase inhibitor, namely elastatinal (produced by actinomycetes). Only crude extracts from isolate SH-82 (*Micromonospora fluostatini*) exhibited sufficient inhibition of elastase activity (Figure 1). The ethyl acetate extract (SH-82-EA-SM) showed the highest inhibition by 32.5% at 100 µg/mL.

#### 2.3.2. Anti-Tyrosinase Activities

**Figure 2:**
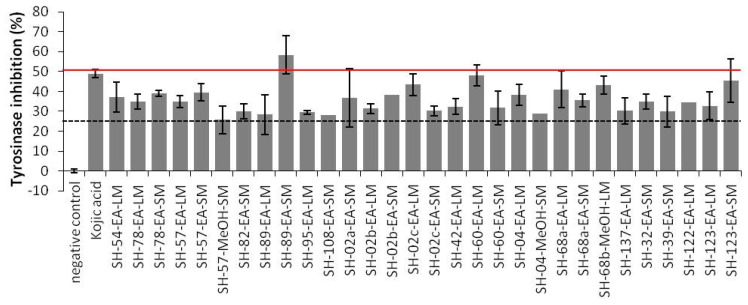
Percentage inhibition of tyrosinase activity by selected microbial crude extracts. Only the most active ones are shown. The code per isolates is outlined in Tables S1–S3. Bars, ±SD; *n* ≥ 2.

Crude extracts were tested on purified mushroom tyrosinase at the concentration of 150 µg/mL for tyrosinase inhibition activity. Their activities were compared to the commercially available tyrosinase inhibitor kojic acid. The screening revealed 29 extracts from 20 different isolates (nine *Bacillus*, five *Micromonospora*, two *Salinispora*, one *Aspergillus*, one *Chaetomium* and two Thermoactinomycetaceae) with moderate tyrosinase inhibitory activities (25–50% inhibition) (Figure 2). Only one extract from SH-89 isolate (*Micromonospora citrea*) exerted significant anti-melanogenic properties, as it showed 58.33% tyrosinase inhibition. In spite of the moderate inhibitory activity exhibited by these isolates, they represent a promising source for the discovery of new anti-melanogenic agents.

#### 2.3.3. Catalase Activities

**Figure 3:**
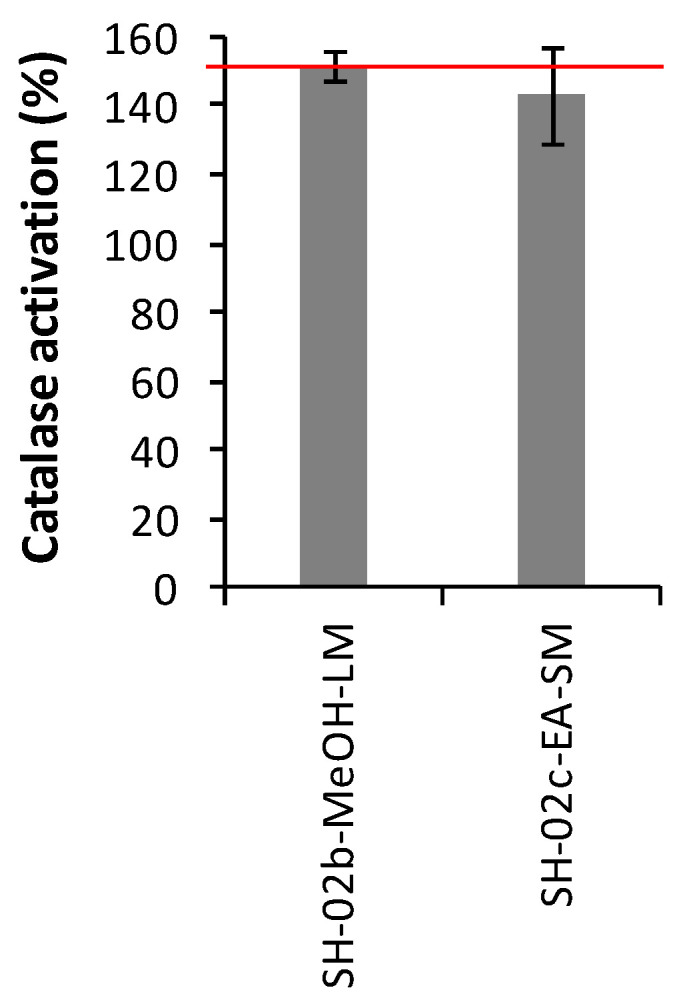
Activation average of catalase by selected microbial crude extracts. Only the most active extracts are shown. The code per isolates are outlined in Table S2. Bars, ±SD; *n* = 4.

All extracts were screened on purified catalase, at the concentration of 10 µg/mL, in order to assess their antioxidant properties through the catalase activation. Four extracts (SH-02a-MeOH-SM, SH-02b-MeOH-LM, SH-02c-EA-SM, SH-02a-MeOH-SM) obtained from *Bacillus* strains exhibited promising antioxidant capability (activation average > 150). These extracts were selected for a secondary screening, leading to the validation of SH-02b-MeOH-LM (*B. paralicheniformis*) and SH-02c-EA-SM (*B. paralicheniformis*) as catalase activators (Figure 3).

#### 2.3.4. Sirtuin 1 Activities

**Figure 4:**
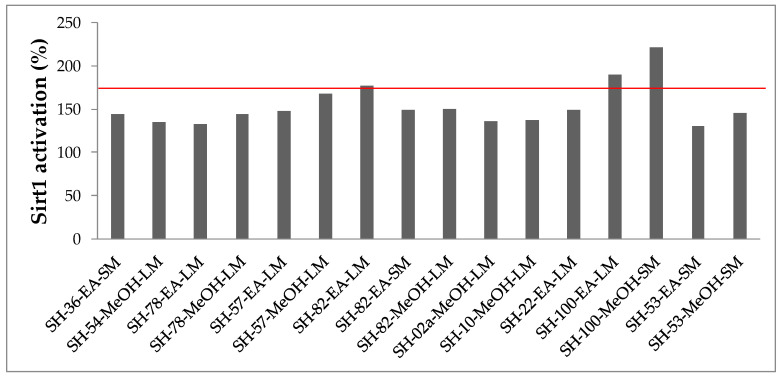
Activation rates of sirtuin 1 by selected microbial crude extracts. Only the most active extracts are indicated. The codes of the isolates are outlined in Tables S1–S3.

Activation of Sirtuin 1 by crude extracts was assessed on purified Sirtuin 1, at the concentration of 100 µg/mL, and expressed as an activation average in comparison to a reference compound (Figure 4). Of the 118 crude extracts tested, 13 extracts activated Sirt1; activation ranged between 170% and 130%. The most potent activators of Sirt1 activity were exhibited by SH-82 (*Micromonospora fluostatini*) and SH-100 (*Bacillus licheniformis*) isolates extracts. These three extracts (SH-82-EA-LM = 177%; SH-100-EA-LM = 190% and SH-100-MeOH-SM = 222%) increased Sirt1 activity with an activation average beyond 170%. During this primary screening, 10 strains were identified as producers of secondary metabolites capable of increasing the activity of Sirtuin 1. The most active ones are SH-82 (*Micromonospora fluostatini*) and SH-100 (*Bacillus licheniformis*). Three more strains of the *Bacillus* genus (*B. paralicheniformis*) gave moderate activities (SH-02a: 136%, SH-10: 137%, SH-22: 149%) along with two *Salinispora* strains (SH-54: 135%, SH-78: 132% and 144%), 2 *Micromonospora* strains (SH-36: 144%, SH-57: 148% and 168%) and one fungi of the *Nigrospora* genus (SH-53: 130% and 146%). Given the scarcity of naturally occurring sirtuin-activating compounds, these strains represent an interesting source for new STACs.

#### 2.3.5. Anti-CDK7 Activities

The microbial crude extracts activity was assessed for inhibition of CDK7 activity in a dose dependent way against purified CDK7 and was compared to the reference compound staurosporine (produced by actinomycete such as *Salinispora*). Screening of all extracts for anti-CDK7 properties revealed three extracts from three different strains of *Salinispora arenicola* (SH-45, SH-54 and SH-78) with appreciable CDK7 inhibitory activities at the highest and medium concentrations tested (0.033 and 0.0033 µg/mL) (Table 2).

#### 2.3.6. Anti-Fyn Kinase Activities

Anti-Fyn activity was assessed on a purified protein Fyn in a dose dependent manner and was compared to the reference compound staurosporine. Nine strains were found to produce secondary metabolites with Fyn kinase inhibitory activities and ten anti-Fyn kinase extracts were identified (Table 3). The most actives were SH-78 *Salinispora arenicola* (SH-78-EA-SM) and SH-04 *Bacillus licheniformis* (SH-04-EA-SM) strains which inhibited Fyn activity at all three tested concentrations. Two more *S. arenicola* strains (SH-45 and SH-54) yielded crude extracts with good inhibitory activities at the highest and medium concentrations, as well as the *Rhodococcus* strain (SH-115), three more *B. licheniformis* strains (SH-68a, SH-68b and SH-116a) and the fungal strain *Chaetomium globosum* (SH-123).

#### 2.3.7. Anti-Proteasome Activities

No dose-response was observed for this bioassay. The active crude extracts exhibited inhibitory activities only at the highest concentration tested (0.033 µg/mL). Of the 118 crude extracts, only five were active, corresponding to three *Salinispora arenicola* strains (SH-45, SH-54 and SH-78) and one *Bacillus licheniformis* strain (SH-99).

In short, 118 microbial crude extracts were screened against a set of molecular targets with potential pharmaceutical and cosmeceutical applications in the field of aging, for the identification of “hit” extracts. There were both quantitative and qualitative differences in the observed biological activities. Variations were observed not only with respect to the isolates but also according to the growth media (cultural conditions) and the extractive solvents used. Fifty-four of the 118 crude extracts (46%) were active in at least one bioassay (anti-elastase, anti-tyrosinase, anti-CDK7, anti-Fyn kinase, anti-proteasome, increased catalase or sirtuin 1 activities). Remarkably, anti-tyrosinase activity was the most common bioactivity. In total, 25% of the crude extracts (from 20 strains: bacteria and fungi) inhibited at least 25% of the tyrosinase activity, whereas only 1.7% of the extracts (from one *Micromonospora* strain: SH-82) inhibited at least 25% of elastase activity. Furthermore, 8.5% of the extracts (from nine strains) inhibited in a dose-depend manner the activity of the Fyn-kinase, while only 2.5% of the extracts (from three *Salinispora* strains) inhibited the activity of CDK7 in a dose-dependent manner. Of the crude extracts, 13.6% (10 strains) were found to increase Sirtuin 1 activity, with an activation average of at least 130%, whilst only 1.7% of the extracts (from four *Bacillus* strains) were found to activate catalase with an activation average of at least 150%. Extracts of only *Salinispora* and *Bacillus* strains (4%) were found to be active against the proteasome at the highest concentration tested. Although some of the isolates showed different biological activities, almost half of them exhibited unique biological activity. *Bacillus paralicheniformis* (SH-42, SH-60), *Micromonospora* (SH-89, SH-95, SH-108), *Aspergillus* (SH-122) as well as Thermoactinomycetaceae (SH-32, SH-39) strains were found to inhibit only tyrosinase activity. SH-36 (*Micromonospora chokoriensis*), SH-100 (*Bacillus licheniformis*), SH-53 (*Nigrospora aurantiaca*), SH-10 and SH-22 (*Bacillus paralicheniformis*) were active only on Sirtuin 1. SH-115 (*Rhodococcus nanhaiensis*) and SH-116 (*Bacillus licheniformis*) inhibited solely the activity of Fyn kinase while SH-99 (*Bacillus licheniformis*) inhibited the proteasome activity. It is also interesting to note that only SH-82 (*M. fluostatini*) inhibited elastase, while solely *B. paralicheniformis* (SH-02a, SH-02b, SH-02c) and *B. berkeleyi* (SH-137) strains succeeded in stimulating catalase activity. All tested isolates, except one of *Bacillus* strain (SH-46), showed some bioactivity, suggesting the presence of bioactive compounds with potential anti-aging activity.

**Figure 5:**
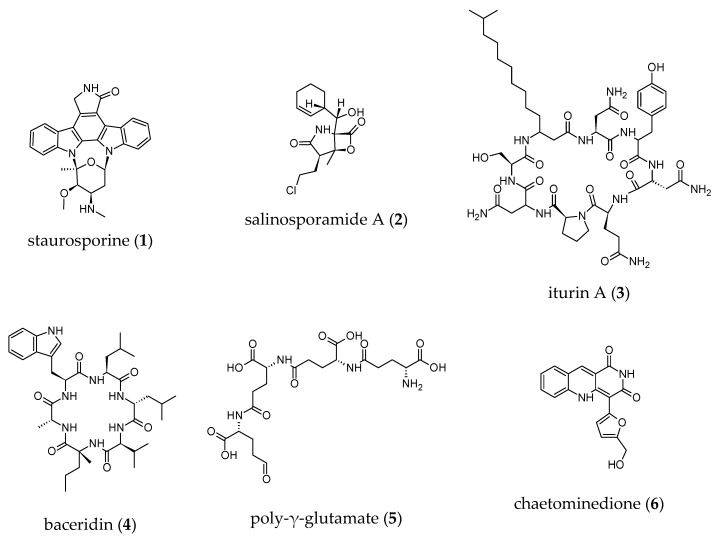
Structures of known microbial secondary metabolites with anti-aging activities isolated from *Bacillus*, *Chaetomium*, *Micromonospora* and *Salinispora* genera.

Most of the active strains isolated in this study belong to genera (*Salinispora*, *Micromonospora*, *Bacillus*, *Aspergillus* and *Chaetomium*), which are well known to be prolific metabolite producers. Biological activities in the natural products field are mainly focused on antimicrobial and anticancer properties. Nonetheless, some selective activities include anti-tyrosinase/anti-elastase activities, kinase and proteasome inhibition. Some compounds isolated from *Salinispora*, *Micromonospora*, *Bacillus* and *Chaetomium* with reported anti-aging activities are presented in Figure 5. For example, the genera *Micromonospora* and *Salinispora* are producers of staurosporine (**1**), a non-selective protein kinase inhibitor, and staurosporine derivatives. These compounds showed cytotoxic activity against a wide range of cancer cell lines [91]. Dereplication of the most active extract from *Salinispora* strain (SH-78) by low resolution mass spectrometry indicated the presence of staurosporine (**1**). The genus *Salinispora* (*S. tropica* and *S. pacifica*) also produce a potent proteasome inhibitor, salinosporamide A (**2**), one of the most promising anticancer agents, currently used in clinical trials for the treatment of multiple myeloma [48]. These compounds might explain the inhibitory activities against protein kinases (CDK7 and Fyn) and proteasome exhibited by *Salinispora* strains. However, to date, compounds in the salinosporamide class were not isolated from *S. arenicola* strains, as the production of this class of compounds appears to be very low in this species [92]. Interestingly, several *Micromonospora* and *Salinispora* strains exhibited anti-tyrosinase activity, while one *Micromonospora* strain showed anti-elastase activity. To our knowledge, no inhibitors of elastase or tyrosinase have been reported from *Micromonospora* or *Salinispora* genera. *Bacillus* species, of which several strains were isolated in this study, increased catalase activity and exhibited anti-tyrosinase, anti-Fyn kinase and anti-proteasome activities. Kinase and proteasome inhibitors with anticancer activity along with some anti-melanogenic compounds were reported from *Bacillus* strains (*Bacillus* spp.) [93,94]. Such an example is iturin A (**3**) (that is well-known for its strong anti-fungal activity) which inhibited protein kinases (MAPK and Akt) as well as baceridin (**4**), a new proteasome inhibitor [95,96]. The iturins are mainly produced by members of the *Bacillus subtilis* group; however, the production of iturins is strongly associated with *B. amyloliquefaciens* [57]. Poly-γ-glutamate (**5**), a natural polymer produced by different bacterial strains including *B. licheniformis*, exhibited strong anti-tyrosinase activity while increasing the catalase activity [93,97]. *Aspergillus* and *Chaetomium* strains (SH-122 and SH-123) were found to inhibit tyrosinase activity. Some fungal metabolites have been identified and reported for their inhibitory activity against tyrosinase. Kojic acid, for example, is produced by many species of *Aspergillus* and extensively used as a skin-whitening agent [39,98]. Curiously, only one isolate of *Chaetomium globosum* had been found to be a low-level producer of kojic acid [99]. *Chaetomium* strain also exhibited a Fyn kinase inhibitory activity that might be explained by the production of chaetominedione (**6**), a tyrosine kinase inhibitor isolated from an algicolous marine specimen of *Chaetomium* sp. [100]. The most remarkable result to emerge from our data was the identification of several crude extracts (16) from *Micromonospora*, *Salinispora*, *Bacillus* and *Nigrospora* strains that increased Sirtuin 1 activity. To date and to the best of our knowledge, few marine natural products or extracts that activate Sirtuin 1 have been reported. Conversely, some marine natural products with anti-Sirtuin 1 activity have been identified. As crude extracts were tested for biological activities, the chemical nature of the bioactive compounds was, up to now, unknown. To gain insight into the metabolic profile of these strains, the active crude extracts will be submitted to liquid chromatography-high resolution mass spectrometry (LC-HRMS/MS) analysis to assess strains with potential to produce novel/known bioactive metabolites. Furthermore, prior to secondary metabolites isolation, fermentation conditions will be optimized in order to improve the yield of produced bioactive metabolites.

**Figure 6:**
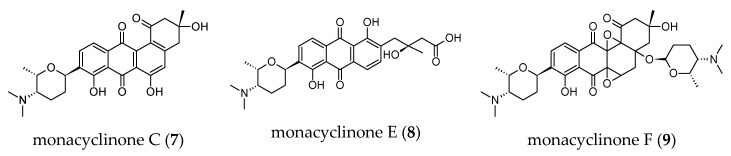
Chemical structures of compounds isolated from *Streptomyces* sp. derived from *S. ruetzleri*.

Another aspect of our future work will address strategies to activate cryptic genes with the aim of identifying new natural products. Indeed, investigation into the microbiome of Australian marine sponges by deep sequencing of NRPS (nonribosomal peptide synthetase) and PKS (polyketide synthase) genes found both NRPS and PKS biosynthetic pathways within *Scopalina* sp. microbiomes [101]. They further revealed within the microbiome of *Scopalina* sp. a great diversity of KS (PKS), the majority of which were trans-AT type and C (NRPS) domains. Trans-AT (*trans*-acyltransferase) type KS domains are an important group of PKS enzymes as they often lead to unique chemistry properties. Importantly, they identified the existence of novel biosynthetic pathways within these sponges, suggesting an untapped resource for natural products discovery. Products of PKS and NRPS pathways or hybrids of both represent a major class of the biologically active microbial natural products of interest to human health and industry. Surprisingly, few bioactive compounds were reported from *Scopalina* spp. In 2015, Vicente and co-workers reported the isolation of six new angucyclinone (aromatic polyketide) derivatives from a *Streptomyces* strain associated with *S. ruetzleri* [102]. Monacyclinone C (**7**) and E (**8**) exhibited moderate cytotoxic activities against rhabdomyosarcoma cancer cells, while monacyclinone F (**9**) showed the highest cytotoxic activity along with antibacterial property (Figure 6). This study represents the first report of the isolation of complex bioactive secondary metabolites from the genus *Scopalina*.

Known to produce species-specific metabolites, studies have found site-specific secondary metabolite gene clusters in *Salinispora* strains collected at different locations. Exploring the secondary metabolism of the strains recovered in this study would be interesting for the discovery of new bioactive natural products. Furthermore, it is now accepted that even well-studied taxa like *Micromonospora* can harbor a wealth of biosynthetic pathways for which the products have yet to be discovered. Interestingly, some of the *Micromonospora* isolates recovered during our work belong to species barely investigated for secondary metabolites production (SH-36 *M. chokoriensis*, SH-89 *M. citrea*, SH-108 *M. endophytica*, SH-82 *M. fluostatini*, SH-95 *M. tulbaghiae*). Moreover, to the best of our knowledge there is no report of secondary metabolites isolated from *Nigrospora aurantiaca* (SH-53) and *Rhodococcus nanhaiensis* (SH-115). The occurrence of PKS and NRPS genes in actinomycetes, *Bacillus* and fungi further supports the high potential of microorganisms associated with the marine sponge *Scopalina hapalia* to produce interesting secondary metabolites. The idea that one microbial strain producing secondary metabolites often has the potential to produce various compounds is now well accepted. In order to reveal the chemical diversity of these strains, alteration of easily accessible cultivation parameters (media composition, adsorbent resins, pH, and temperature) will be carried out. The manipulation of the fermentation conditions, known as the OSMAC (One Strain Many Compounds) approach, represents an easy and effective way of activating silent or poorly expressed biosynthetic pathways [103].

## 3. Materials and Methods

### 3.1. Sponge Collection

*Scopalina hapalia* (ML-263) was collected in May 2013 by scuba diving at the depths of 2–10 m around the southeast coasts of Mayotte (Kani tip, Global Positioning System (12°57.624’ S; 45°04.697’ E)). The sponge sample was levered off with thin-bladed knife to prevent damage, transferred to a plastic bag and kept at -20 °C before being transported to the laboratory. For identification, a voucher specimen was preserved in 80% ethanol. The taxonomic identification was performed by Nicole de Voogd and voucher specimens were deposited at Naturalis Biodiversity Center, Leiden the Netherlands as RMNH POR.8332 and RMNH POR.8376.

### 3.2. Targeted 16S rRNA Gene Sequencing / Next Generation Sequencing

Sponge specimen was cut into pieces of ca. 1 g (1 cm^3^), rinsed in ethanol 70% then in sterile artificial seawater (ASW) (Sea salts (Sel Instant Ocean, Aquarium système, Sarrebourg, France) 33 g/L) [104]. Genomic DNA was extracted from small pieces of sponge using a commercial genomic DNA extraction kit (Qiagen, Hilden, Germany) [105], as per manufacturer’s instructions. DNA extract was visually checked for quality by agarose gel electrophoresis. The purity of the extraction was assessed, and the quantification was undertaken using a NanoDrop spectrophotometer (Thermo Fisher, Waltham, MA, USA). The 16S rRNA gene amplification, sequencing and taxonomic affiliation were performed by Genoscreen (Lille, France) according to their methodology Metabiote®. Briefly, 5 ng of genomic DNA sample were used for libraries preparation, and sequencing was performed using the Illumina MiSeq “paired-end” 2 × 300 bp technology, according to the Metabiote® protocol established by Genoscreen. The hypervariable regions V1–V3, V3–V4 and V4–V5 found in bacteria and archaea were targeted using specific primers. Data preprocessing and analysis of the sequence data were carried out using the Metabiote® v. 2.0 pipeline (Genoscreen, Lille, France), partially based on the QIIME v. 1.9.1 [106]. Raw forward and reverse sequence reads were assembled into contigs using 30 bp coverage (overlapping regions) and 97% sequence identity as parameters for merging with FLASH (Fast Length Adjustment of SHort reads) [107]. Chimeric sequences were identified and discarded by using a Genoscreen program based on Usearch 6.1. Filtered sequences were clustered into Operational Taxonomic Units (OTUs) at 97% sequence similarity using the algorithm Uclust v. 1.2.22q based on an open-reference OTU strategy [108]. The most abundant sequence for each OTU was used as reference and aligned to the Greengenes database v. 13.8 (greengenes.secondgenome.com). The phyla- and genus-level affiliation of the sequences was validated using the Ribosomal Database Project Classifier v. 2.2 [109].

### 3.3. Alpha Diversity Analysis

Bacterial richness and diversity estimators (observed OTUs, Chao1 and Shannon) were calculated with the script alpha_diversity.py QIIME v. 1.9.1 [110,111].

### 3.4. Microbial Isolation

Sponge specimen was cut into pieces of ca. 1 g (1 cm^3^), rinsed in ethanol 70% and then in sterile ASW. After surface sterilization, the sample was thoroughly homogenized in a sterile mortar then transferred in a 50 mL Falcon vial containing 10 mL of sterile ASW. Two options were selected for the microbial isolation. Protocol 1 (P1): To allow the dissemination of the maximum revivable strains, the homogenate was diluted in ten-fold series (10^−1^, 10^−2^ and 10^−3^) and subsequently plated out on agar plates. Protocol 2 (P2): To eliminate fast growing/heat sensitive strains and favor the slow growing ones, the same protocol was repeated with a heat-shock pretreatment of the homogenate (30 min, 50 °C) before dilution (10^−1^, 10^−2^ and 10^−3^). Eight different media were used for the isolation of microorganisms A1BFe+C (starch (BD Difco^TM^, Le Pont de Claix, France) 10 g, yeast extract (BD Bacto™, Le Pont de Claix, France) 4 g, peptone (BD Bacto™, Le Pont de Claix, France) 2 g, CaCO_3_ (Carlo Erba, Val de Reuil, France) 1 g, Fe_2_(SO_4_)_3_ (Carlo Erba, France) 40 mg, KBr (Carlo Erba, France) 100 mg, sea salts 30 g), LB (tryptone (Sigma Aldrich, St. Louis, MI, USA, Steinheim, Germany) 10 g, Yeast Extract 5 g, NaCl (Fisher Scientific Labosi, Elancourt, France) 10 g), Marine Broth (MB) (BD Difco^TM^, Le Pont de Claix, France), MYA2 (malt extract (BD Difco™, Le Pont de Claix, France) 20 g, yeast extract 1 g, sea salts 30 g), Potato Dextrose Broth (PDB) (BD Difco^TM^, Le Pont de Claix, France), R2A (Fisher Scientific, Waltham, MA, USA), SCAM (maize starch (Fisher Chemical, Loughborough, UK) 10 g, casein (VWR Chemicals, Leuven, Belgium) 1 g, sea salts 30 g), SFM (soybean flour (La Vie Claire, Montagny, France) 20 g, mannitol (Carlo Erba, Val de Reuil, France) 20 g). All media contained Difco^TM^ Bacto agar (20 g/L) and were prepared in 1 L of purified water with pH adjusted to 7.2 ± 0.2, except for SFM. This last medium was prepared with tap water and no pH adjustment. The inoculated plates were incubated at 28 °C for 10 weeks. Distinct colony morphotypes were picked and re-streaked until visually free of contaminants. Isolates were inoculated on A1BFe+C for putative actinomycetes, on LB for bacteria and MYA2 for fungi. The isolates were maintained on plates at 4 °C for short-term conservation. For long-term strain collection, the microorganisms were stored at −80 °C in a cryoprotectant medium (skimmed milk (Régilait, Macon, France) 10% (*w*/*v*), glycerol (Carlo Erba, Val de Reuil, France) 10% (*v*/*v*) and sea salts 33 g/L). Bacterial strains were sorted into groups according to their morphological characteristics. Twelve putative actinomycetes and three filamentous fungi were selected for molecular identification and secondary metabolites production. Fifteen other bacteria types giving mucoid/smooth colonies adhering to the agar surface and/or showing cocci/bacilli morphology were included in this study.

### 3.5. Molecular Identification

Selected bacterial strains were cultured on A1BFe+c medium for two to 14 days and then were sent to Genoscreen (Lille, France). Targeted 16S rRNA gene amplification (V1–V3 and V3–V5) and sequencing were performed by Genoscreen. The obtained contigs were compared to EzBioCloud 16S database (www.ezbiocloud.net, accessed on 31 May 2019) for species-level identification, using sequence similarity searches [112].

The fungal strains were cultured on PDA medium and then sent to the Westerdijk Fungal Biodiversity Institute (Netherland) for identification. The PDA medium consisted of PDB supplemented with agar at the final concentration of 2%. On arrival, the strains were cultivated on malt extract agar (MEA) and dichloran 18% glycerol agar (DG18). DNA was extracted from one MEA plate after an incubation period of three days in the dark at 25 °C, using the Qiagen DNeasy Ultraclean™ Microbial DNA Isolation Kit (Qiagen, Germany). For strain SH-53, fragments containing the Internal Transcribed Spacer 1 and 2 regions including the 5.8S rDNA (ITS) and a partial β-tubulin gene (*BenA*) were amplified and sequenced. For strain SH-122, fragments containing a partial β-tubulin gene (*BenA*) and fragments containing a partial calmodulin gene (*CaM*) were amplified and sequenced. For strain SH-123, fragments containing the Internal Transcribed Spacer 1 and 2 regions including the 5.8S rDNA (ITS) and a partial ß-tubulin gene (*BenA*) were amplified and sequenced. The primers used were: ITS (SH-53 and SH-123): LS266 (GCATTCCCAAACAACTCGACTC) and V9G (TTACGTCCCTGCCCTTTGTA), *BenA* (SH-53 and SH-122): Bt2a (GGTAACCAAATCGGTGCTGCTTTC) and BT2b (GGTAACCAAATCGGTGCTGCTTTC), *CaM*: CMD5 (CCGAGTACAAGGARGCCTTC) and CMD6 (CCGATRGAGGTCATRACGTGG), *BenA* (SH-123): T1 (AACATGCGTGAGATTGTAAGT) and Tub4RD (CCRGAYTGRCCRAARACRAAGTTGTC). The PCR fragments were sequenced in both directions with the primers used for PCR amplification using the ABI Prism® Big DyeTM Terminator v. 3.0 Ready Reaction Cycle sequencing Kit (Thermo FisherFisher, USA). Samples were analyzed on an ABI PRISM 3700 Genetic Analyzer and contigs were assembled using the forward and reverse sequences with the program SeqMan from the LaserGene package. The sequences were compared on GenBank using BLAST (www.ncbi.nlm.nih.gov) and in the in-house sequence database of Westerdijk Fungal Biodiversity Institute.

### 3.6. Extracts Preparation

Thirty strains were selected based on their affiliation to taxa known to produce bioactive secondary metabolites with biotechnological interests. Prior to fermentation, each of the selected isolates were inoculated on two Petri plates (Ø 9 cm, Nunc™ Thermo FisherFisher, USA) containing MBA (Marine Broth supplemented with 2% agar) and incubated for 5–17 days depending on their growth rate at 28 °C. About 5 mL of ASW were spread on the agar layer, the culture was then scratched and transferred in a 50 mL Falcon vial containing 45 mL of ASW in order to prepare the inoculum. Then, 10 mL of the homogenate was used to inoculate a 2 L Erlenmeyer flask containing 1 L of MB mixed with 30 g/L of XAD-16 resin (Sigma, St. Louis, USA; Steinheim, Germany) (liquid-state fermentation coupled to in-situ solid-phase extraction) [113]. The remainder homogenate was mixed with 35 g of XAD-16 resin and uniformly spread on a large-surface Petri dish (25 × 25 cm, Nunc™ Thermo Fisher, USA) containing MBA (solid-state fermentation coupled to solid-phase extraction) [114]. The liquid cultures were grown for 5 days at 28 °C, then the biomass and the resins were recovered by Buchner filtration (filter paper Whatman® grade 4, Ø 110 mm). The solid cultures were grown for 15 days at 28 °C; then the mix of biomass/resins was peeled off. All the biomass/resins mixes were washed with distilled water, dried under vacuum on Buchner filter and successively extracted with ethyl acetate (EA) (Carlo Erba, France) and methanol (MeOH) (Carlo Erba, France). The solvents were removed by evaporation; the residues were weighed and used for biological assays.

### 3.7. Bioassays

Bioassays against selected targets involved in age-related diseases and disorders (elastase, tyrosinase, catalase, sirtuin 1, CDK7, Fyn and proteasome) were carried out as follows.

#### 3.7.1. Elastase Activity Assay

Elastase enzyme activity was evaluated using elastase from porcine pancreas (PPE) type IV and *N*-succinyl-Ala-Ala-Ala-p-nitroanilide as substrate, as previously described [115]. The amount of released p-nitroaniline, which was hydrolyzed by elastase, was measured spectrophotometrically at 405 nm. The reaction mix was constituted of 70 µL Trizma-base buffer (50 mM, pH 7.5), 10 µL of the extract tested (the final concentration of the extracts was 100 µg/mL) and 5 µL of PPE (0.4725 U/mL), in a 96-well microplate. The samples were incubated for 15 min in room temperature, avoiding light exposure. Subsequently, 15 µL from 0.903 mg/mL *N*-succinyl-Ala-Ala-Ala-p-nitroanilide were added and the samples were incubated at 37 °C for 30 min. Then, the absorbance of p-nitroaniline production was measured in the reader Infinite 200 PRO series (Tecan). Elastatinal was used as positive control, whereas the negative control contained the Trizma-base buffer and the substrate. Experiments were performed in duplicates. The reagents of the assay were purchased from Sigma-Aldrich. The percentage of elastase inhibition was calculated as follows:

Inhibition (%) = {[(Abs control-Abs control’s blank) − (Abs sample-Abs sample’s blank)] / (Abs control-Abs control’s blank)]} ∗ 100,

where Abs control is the absorbance of the elastase in Trizma base buffer, sample solvent and substrate, and Abs sample is the absorbance of the elastase in Trizma base buffer, extract or elastatinal and substrate. Blank experiments were performed for each sample with all the reagents except the enzyme.

#### 3.7.2. Tyrosinase Activity Assay

In order to evaluate the inhibitory potency of the extracts against tyrosinase, the oxidation of L-DOPA to dopachrome was determined by an enzymatic method, as previously described [116]. Specifically, in a 96-well microplate, 80 µL of PBS (0.067 M, pH 6.8), 40 µL of the tested extract (the final concentration of the extracts was 150 µg/mL) and 40 µL of mushroom tyrosinase 92 U/mL were mixed and incubated for 10 min at room temperature, avoiding light exposure. Afterwards, 40 µL of 2.5 mM L-DOPA dissolved in PBS buffer were added and the mixture was incubated for 5 min before measurement of dopachrome formation at 475 nm using the reader Infinite 200 PRO series (Tecan, Salzburg, Austria). Kojic acid was used as positive control, whereas the negative control contained PBS and the substrate. Experiments were performed in duplicates. The reagents of the assay were purchased from Sigma-Aldrich. The tyrosinase inhibition percentage was calculated as follows:

Inhibition (%) = {[(Abs control-Abs control’s blank) − (Abs sample-Abs sample’s blank)] / (Abs control-Abs control’s blank)]} ∗ 100,

where Abs control is the absorbance of tyrosinase enzyme in PBS, sample solvent, and substrate and Abs sample is the absorbance of tyrosinase enzyme in PBS, extract or Kojic acid, and substrate. Blanks contained all the aforementioned components except the enzyme.

#### 3.7.3. Catalase Activation Assay

Catalase activity was measured using The Amplex® Red Catalase Assay Kit according to the manufacturer’s instructions (Thermo Fisher Scientific, Waltham, MA, USA). Briefly, 2.5 µL of catalase (final concentration 62.5 mU/mL) first reacts with 5 µL of H_2_O_2_ (final concentration 10 µM) to produce water and oxygen (O_2_). Next 10 µL of the Amplex Red reagent (final concentration 50 µM) reacts with any unreacted H_2_O_2_ in the presence of horseradish peroxidase (HRP) to produce the highly fluorescent oxidation product, resorufin. Primary and secondary screenings were performed respectively in monoplicate and quadruplicate. The fluorescence was measured on a Polar Star Omega (BMG Labtech, Ortenberg, Germany) plate reader. The results are typically plotted by subtracting the observed fluorescence from that of a no-catalase control.

#### 3.7.4. Sirtuin 1 Activation Assay

Sirt1 activity was measured using SIRT 1 Fluorometric Drug Discovery Kit according to the manufacturer’s instructions (Enzo Life Sciences, Farmingdale, NY, USA). Briefly, this assay uses a small lysine-acetylated peptide, corresponding to K382 of human p53, as a substrate. The lysine residue is deacetylated by SIRT1, and this process is dependent on the addition of exogenous NAD+. The assay was carried at 37 °C using Greiner white, small volume 384 well plates. First, 4 µL of substrate Fluor de Lys (final concentration 25 µM) were mixed with 4 µL of extract (stock concentration 10 mg/mL) previously diluted 1/100 in assay buffer and 2 µL of the enzyme were added. After an incubation of 15 min at 37 °C, 10 µL of Developer 1× solution (composed by buffer, developer 5× and Nicotinamide 50 mM) were added and incubated for 45 min at 37 °C. After 45 min, the fluorescence was measured on a Polar Star Omega (BMG Labtech, Ortenberg, Germany) plate reader. The fluorescence generated was proportional to the quantity of deacetylated Lysine (i.e., corresponding to Lysine 382). All measurements were performed in monoplicate and the final DMSO concentration was 0.1%. SIRT1 inhibitors nicotinamide (2 mM), suramin (100 µM), and sirtinol (100 µM) were used to confirm the specificity of the reaction. Calculation of net fluorescence included the substraction of a blank consisting of buffer containing no NAD+ and expressed as a percentage of control.

#### 3.7.5. CDK7 Inhibition Assay

CDK7 activity was evaluated using CDK7 (Crelux construct CZY-3, PC09891). The inhibitory potency of the extracts against CDK7 was determined by using the ADP-Glo Kinase Assay (Promega, Madison, WI, USA) and CDKtide as substrate. The assay was carried out at 22 °C using Corning 4513 white low volume 384 well plates. All measurements were performed in singlicate and the final DMSO concentration was 3.3%. The assay buffer contained 20 mM Hepes pH 7.5, 150 mM NaCl, 10 mM MgCl2. First, 9.5 µL protein dilution (final concentration 300 nM) were mixed with 0.5 µL of either one of three extract dilutions (1, 0.1 or 0.01 mg/mL >> assay end conc. 0.033, 0.0033 or 0.00033 mg/mL) and preincubated for 120 min. Then 5 µL substrate/ATP mix were added (final concentration substrate 30 µM and ATP 125 µM) and the assay was incubated for another 2 h. Afterwards, 5 µL of the 15 µL assay reaction were transferred to new wells and 5 µL ADP Glo Reagent were added to terminate the kinase reaction and deplete the remaining ATP. After 40 min, 10 µL of Kinase Detection Reagent were added to convert ADP to ATP and allow the newly synthesized ATP to be measured using a luciferase/luciferin reaction. After another 40 min, the assay plates were measured in Luminescence mode on a Tecan M1000 plate reader. The light generated, i.e., luminescent signal, was proportional to the ADP concentration produced and was correlated with kinase activity. The IC50 was calculated using XLfit. Staurosporine, which inhibits CDK7, was used as positive control in order to assess the functionality of the assay.

#### 3.7.6. FynB Inhibition Activity

FynB activity was evaluated using FynB wt (Crelux construct CTX4, PC09815-1). The inhibitory potency of the extracts against FynB was determined by using the ADP-Glo Kinase Assay (Promega) and Fyn kinase substrate (Enzo Life Sciences, P215). The assay was carried out at room temperature (22 °C) using Corning 4513 white low volume 384 well plates. All measurements were performed in singlicate and the final DMSO concentration was 3.3%. The assay buffer contained 20 mM Tris pH 8.0, 170 mM NaCl, 10 mM MgCl2. First, 9.5 µL protein dilution (final concentration 200 nM) were mixed with 0.5 µL of either one of three extract dilutions (1, 0.1 or 0.01 mg/mL >> assay end conc. 0.033, 0.0033 or 0.00033 mg/mL) and preincubated for 90 min. Then 5 µL substrate/ATP mix were added (final concentration substrate 10 µM and ATP 100 µM) and the assay was incubated for another 2 h. Afterwards, 5 µL of the 15 µL assay reaction were transferred to new wells and 5 µL ADP Glo Reagent were added to terminate the kinase reaction and deplete the remaining ATP. After 40 min, 10 µL of Kinase Detection Reagent were added to convert ADP to ATP and allow the newly synthesized ATP to be measured using a luciferase/luciferin reaction. After another 40 min, the assay plates were measured in Luminescence mode on a Tecan M1000 plate reader. The light generated, i.e., luminescent signal, was proportional to the ADP concentration produced and was correlated with kinase activity. The IC50 was calculated using XLfit. Staurosporine, which inhibits FynB kinase, was used as positive control in order to assess the functionality of the assay.

#### 3.7.7. Proteasome Inhibition Assay

Proteasome activity was evaluated using yeast proteasome (TUM Groll group). The inhibitory potency of the extracts against yeast proteasome was assessed by using the Fluorescence Intensity Assay and Suc-Leu-Leu-Val-Tyr-AMC as substrate (Enzo Life Sciences, BML-P802-0005). The assay was carried out at room temperature (22 °C) using Corning 4514 black low volume 384 well plates. All measurements were performed in singlicate and the final DMSO concentration was 3.3%. The assay buffer contained 100 mM Tris pH 7.5 and 1 mM MgCl2. First, 9.5 µL protein dilution (final concentration 9 nM) were mixed with 0.5 µL of either one of three probe dilutions (1, 0.1 or 0.01 mg/mL >> assay end conc. 0.033, 0.0033 or 0.00033 mg/mL) and preincubated for 90 min. Then 5 µL substrate mix were added (final concentration 5 µM) and incubated for 60 min. The assay plates were measured in fluorescence mode on a Tecan M1000 plate reader (ex 380 nm, em 460 nm); the IC50 was calculated using XLfit. ONX-0914, which inhibits yeast proteasome, was used as positive control in order to assess the functionality of the assay.

## 4. Conclusions

The sponge-microbial associations from the southwest of Indian Ocean were not extensively explored for their potential to produce clinically relevant bioactive compounds. In this study, 30 isolates belonging to genera *Bacillus*, *Micromonospora*, *Rhodococcus*, *Salinispora*, *Aspergillus*, *Chaetomium*, *Nigrospora* and unidentified genera related to the family Thermoactinomycetaceae were recovered from the marine sponge *Scopalina hapalia* collected in Mayotte. In total, 54 of 118 microbial crude extracts from 29 different strains showed significant bioactivities against age-related molecular targets. These results demonstrate that the selected strains produce secondary metabolites with likely anti-aging activities. Taken together, these findings highlight the ability of marine sponge-associated microorganisms from Mayotte to produce secondary metabolites with cosmeceutical and therapeutic potential. Our marine microbial collection represents an important “eco-friendly” resource for the bioprospection of novel bioactive metabolites.

## Figures and Tables

**Table 1:** Alpha diversity of the microbial community associated with *Scopalina hapalia* as measured by the mean index (observed OTUs, Chao1 and Shannon) ± SD.

Targeted Region	No of Reads (SI 97%)	No. of Quality-Filtered Reads	No. of OTUs	Richness		Diversity
				Observed OTUs	Chao1	Shannon
V1–V3	20806	16353	337	286.74 ± 98.97	308.97 ± 94.37	5.65 ± 0.87
V3–V4	49235	47630	408	315.79 ± 119.56	361.43 ± 118.27	4.04 ± 0.69
V4–V5	39094	37213	355	313.23 ± 103.65	324.04 ± 101.00	5.39 ± 0.82
ITS2	46442	46370	32	26.88 ± 9.18	28.92 ± 9.21	0.52 ± 0.02

SI 97%: sequence similarity cutoff of 97%. OTUs: operational taxonomic units.

**Table 2:** Dose-response inhibition of CDK7 activity by microbial crude extracts. Only the most active extracts are shown. Code of isolates are outlined in Table S1.

Extracts	Dose-Response Inhibition		
	0.033 µg/mL	0.0033 µg/mL	0.00033 µg/mL
SH-45-EA-SM	Yes	Yes	No
SH-54-EA-SM	Yes	Yes	No
SH-78-EA-SM	Yes	Yes	No

**Table 3:** Dose-response inhibition of Fyn activity by microbial crude extracts. Only the most active extracts are shown. Code of isolates are outlined in Tables S1–S3.

Extracts	Dose-Response Inhibition		
	0.033 µg/mL	0.0033 µg/mL	0.00033 µg/mL
SH-45-EA-SM	Yes	Yes	No
SH-54-EA-SM	Yes	Yes	No
SH-78-EA-SM	Yes	Yes	Yes
SH-115-EA-SM	Yes	Yes	No
SH-04-EA-SM	Yes	Yes	Yes
SH-68a-EA-LM	Yes	Yes	No
SH-68b-EA-LM	Yes	Yes	No
SH-116a-EA-SM	Yes	Yes	No
SH-123-MeOH-LM	Yes	Yes	No
SH-123-MeOH-SM	Yes	Yes	No

## Supplementary Materials

The following are available online at https://www.mdpi.com/2076-2607/8/9/1262/s1, Figure S1. Rarefaction curves of observed OTUs for each targeted genomic DNA region, Figure S2. Rarefaction curves of Chao1 Index for each targeted genomic DNA region, Figure S3. Rarefaction curves of Shannon Index for each targeted genomic DNA region. Figure S4. Phylum distribution in *Scopalina hapalia* respective with V1–V3, V3–V4 and V4–V5 data sets. The bars represent the relative abundance of 16S rRNA sequences that were assigned to a given phylum in relation to the total number of sequences in each data set. Table S1. 16S rRNA taxonomic affiliation of 10 of the *Scopalina hapalia* associated actinomycetes. Table S2. 16S rRNA taxonomic affiliation of 17 Bacillales (order) strains isolated from *Scopalina hapalia*. Table S3. Taxonomic affiliation of the 3 fungal isolates from *Scopalina hapalia* after sequencing fragments containing ITS region as well as partial beta-tubulin and calmodulin genes.
