# Supplementary material for: Microorganisms Associated with the Marine Sponge Scopalina hapalia: A Reservoir of Bioactive Molecules to Slow Down the Aging Process
Source: Microorganisms. 2020 Aug 20;8(9):1262. doi: 10.3390/microorganisms8091262 (PMC7570120; doi:10.3390/microorganisms8091262)
Supplement: Supplementary file 1 [file microorganisms-08-01262-s001.pdf]

Supporting Information

Microorganisms Associated with the Marine Sponge *Scopalina hapalia*: a Reservoir of Bioactive Molecules to Slow Down the Aging Process

Charifat Said Hassane [1], Mireille Fouillaud [1], Géraldine Le Goff [2], Aimilia D. Sklirou [3], Jean Bernard Boyer [1], Ioannis P. Trougakos [3], Moran Jerabek [4], Jérôme Bignon [2], Nicole J.de Voogd [5,6], Jamal Ouazzani [2], Anne Gauvin-Bialecki [1,*] and Laurent Dufossé [1,*]

1 Laboratoire de Chimie et Biotechnologie des Produits Naturels, Faculté des Sciences et Technologies, Université de La Réunion, 15 Avenue René Cassin, CS 92003, 97744 Saint-Denis CEDEX 9, La Réunion, France; charifat.said-hassane@univ-reunion.fr (C.S.H.); mireille.fouillaud@univ-reunion.fr (M.F.); jean-bernard.boyer@univ-reunion.fr (J.B.B.); anne.bialecki@univ-reunion.fr (A.G.-B.), laurent.dufosse@univ-reunion.fr (L.D.)

2 Institut de Chimie des Substances Naturelles, CNRS UPR 2301, Université Paris-Saclay, 1, av. de la Terrasse, 91198 Gif-sur-Yvette, France; geraldine.legoff@cnrs.fr (G.L.G.); jerome.bignon@cnrs.fr (J.B.); Jamal.Ouazzani@cnrs.fr (J.O.)

3 Department of Cell Biology and Biophysics, Faculty of Biology, National and Kapodistrian University of Athens, 15784 Athens, Greece; asklirou@biol.uoa.gr (A.D.S.); itrougakos@biol.uoa.gr (I.P.T.)

4 Crelux GmbH, Am Klopferspitz 19a, 82152 Martinsried, Germany; Moran_Jerabek@wuxiapptec.com

5 Naturalis Biodiversity Center, Darwinweg 2, 2333 CR Leiden, The Netherlands; nicole.devoogd@naturalis.nl

6 Institute of Environmental Sciences, Leiden University, Einsteinweg 2, 2333 CC Leiden, The Netherlands

* Correspondence: anne.bialecki@univ-reunion.fr (A.G.-B.) and laurent.dufosse@univ-reunion.fr (L.D.)

Figure S1. Rarefaction curves of observed OTUs for each targeted genomic DNA region. 2
Figure S2. Rarefaction curves of Chao1 Index for each targeted genomic DNA region...... 2
Figure S3. Rarefaction curves of Shannon Index for each targeted genomic DNA region. . 3
Figure S4. Phylum distribution in *Scopalina hapalia* respective with V1-V3, V3-V4 and V4-V5 data sets. The bars represent the relative abundance of 16S rRNA sequences that were assigned to a given phylum in relation to the total number of sequences in each data set. ... 4

Table S1. 16S rRNA taxonomic affiliation of 10 of the *Scopalina hapalia* associated actinomycetes. 5
Table S2. 16S rRNA taxonomic affiliation of 17 Bacillales (order) strains isolated from *Scopalina hapalia*.. 5
Table S3. Taxonomic affiliation of the 3 fungal isolates from *Scopalina hapalia* after sequencing fragments containing ITS region as well as partial beta-tubulin and calmodulin genes.. 5

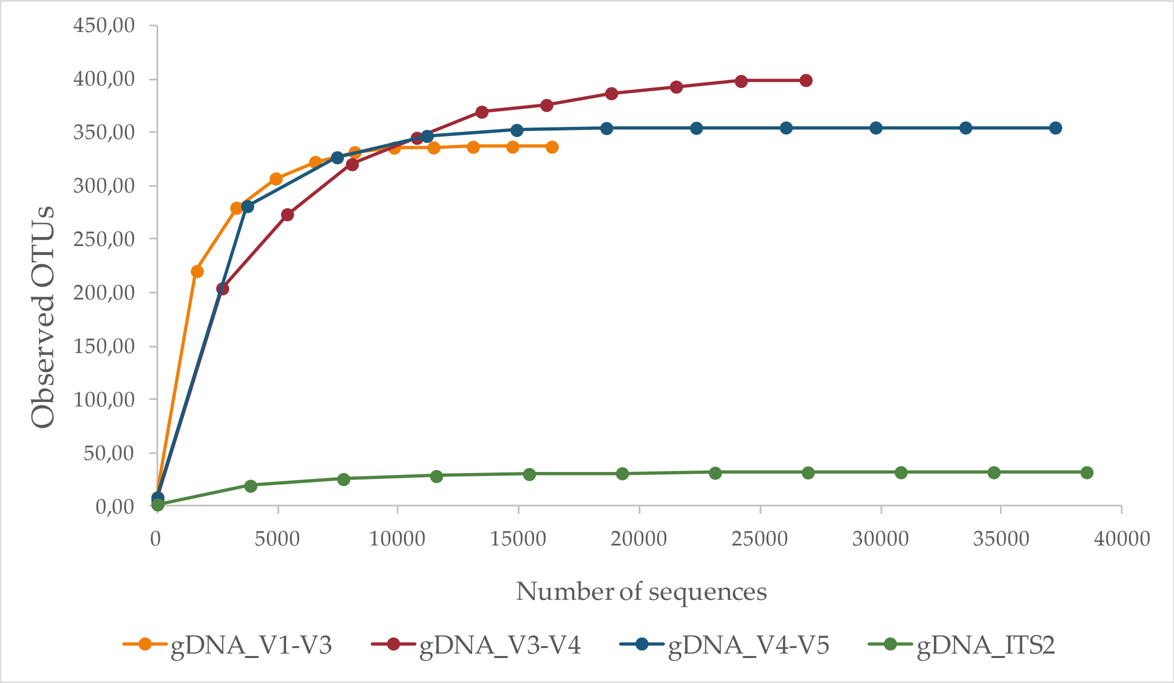

Figure S1. Rarefaction curves of observed Operational Taxonomic Units (OTUs) for each targeted genomic DNA region.

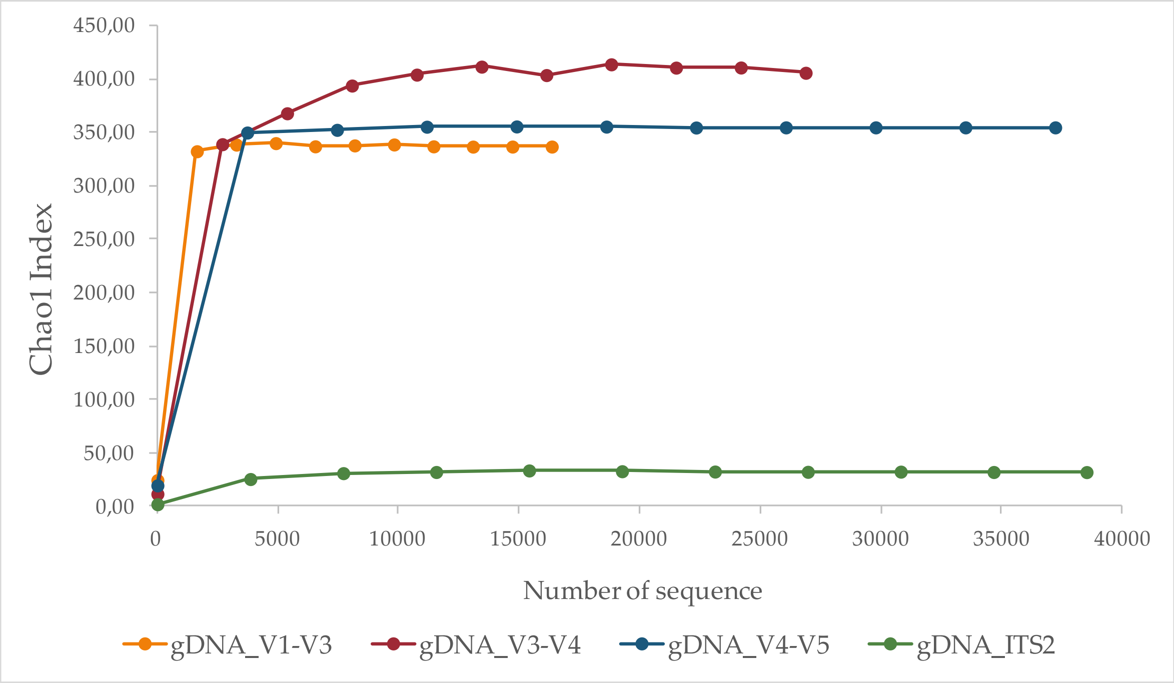

Figure S2. Rarefaction curves of Chao1 Index for each targeted genomic DNA region.

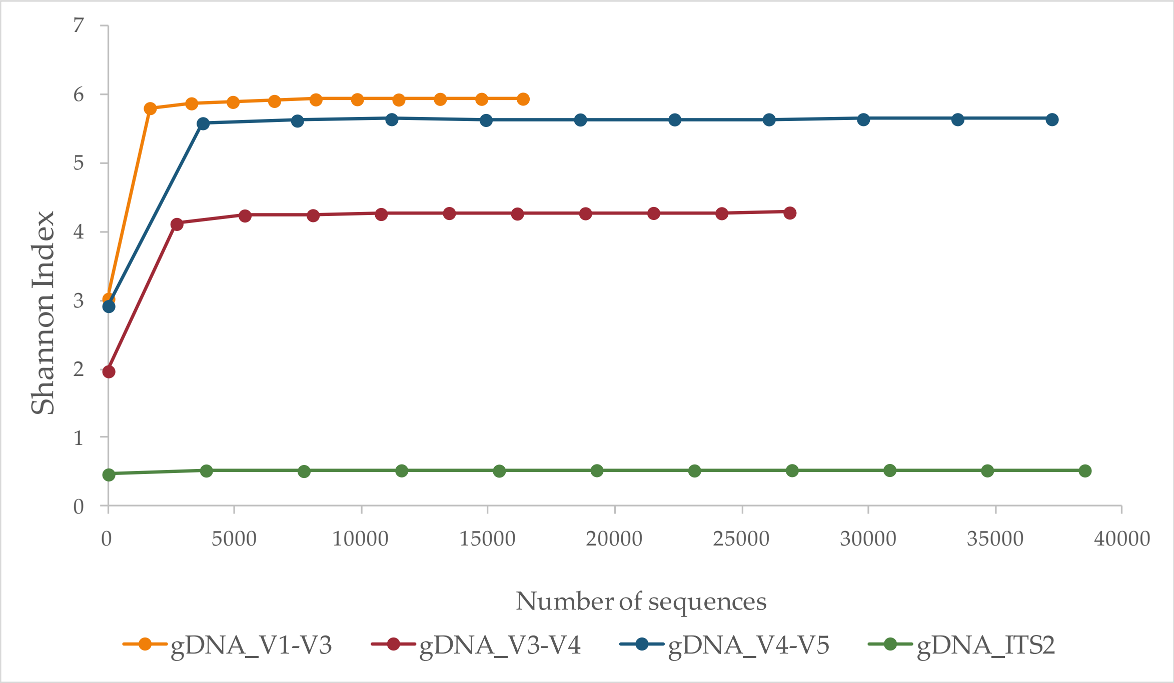

Figure S3. Rarefaction curves of Shannon Index for each targeted genomic DNA region.

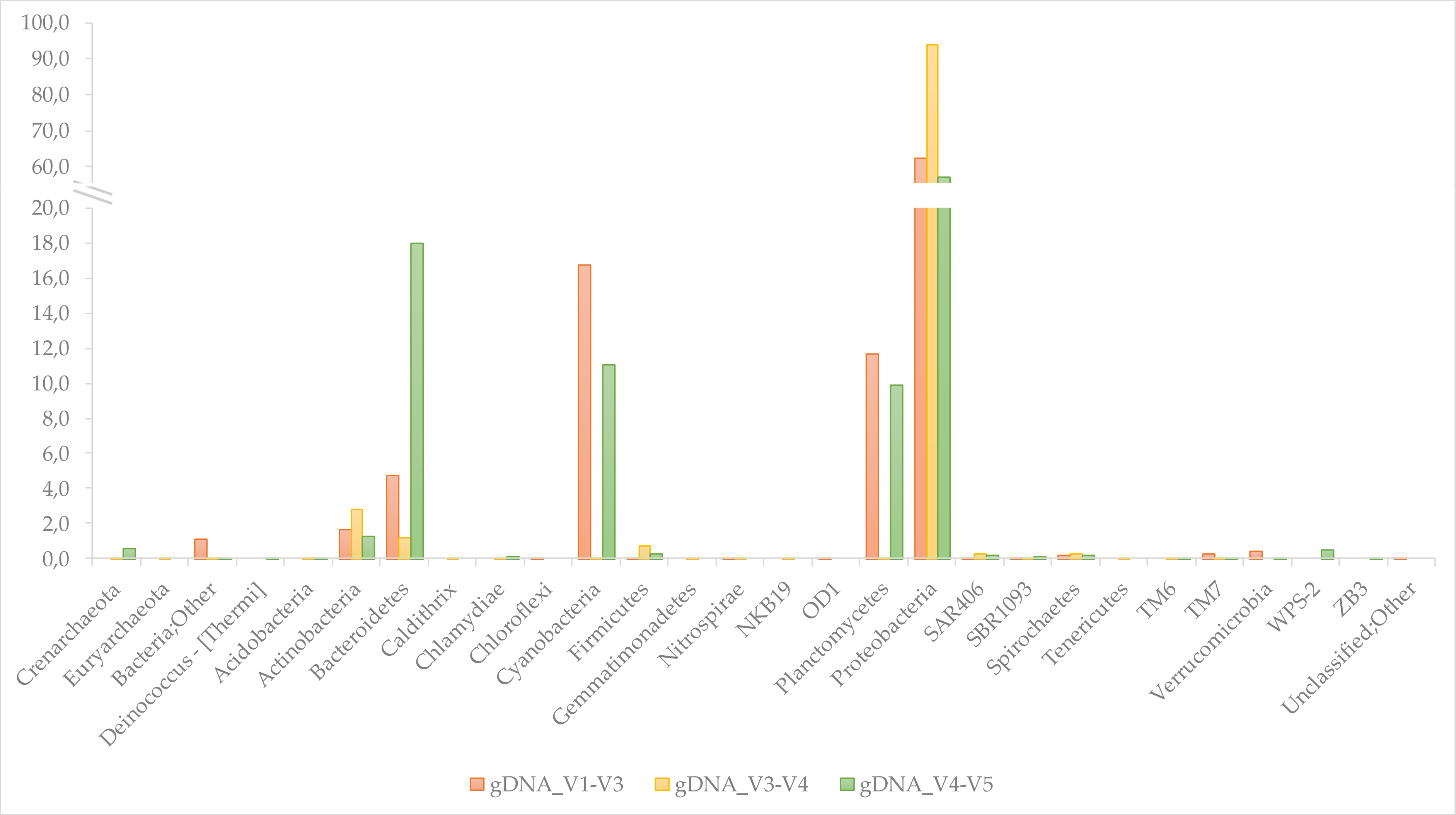

Figure S4. Phylum distribution in *Scopalina hapalia* respective with V1-V3, V3-V4 and V4-V5 data sets. The bars represent the relative abundance of 16S rRNA sequences that were assigned to a given phylum in relation to the total number of sequences in each data set.

Table S1. 16S rRNA taxonomic affiliation of 10 of the *Scopalina hapalia* associated actinomycetes.

Isolate	Isolation Medium	Class	Closest relative in EzBioCloud (Access Number)	Identity (%) [a]
SH-36	R2A	Micromonosporales	*Micromonospora chokoriensis* (LT607409)	100.0
SH-45	SCAM	Micromonosporales	*Salinispora arenicola* (AY040619)	100.0
SH-54	MBA	Micromonosporales	*Salinispora arenicola* (AY040619)	100.0
SH-57	A1BFe+c	Micromonosporales	*Micromonospora echinospora* (LT607413)	99.6
SH-78	MBA	Micromonosporales	*Salinispora arenicola* (AY040619)	100.0
SH-82	MBA	Micromonosporales	*Micromonospora fluostatini* (LC033898)	99.2
SH-89	SCAM	Micromonosporales	*Micromonospora citrea* (FMHZ01000002)	99.5
SH-95	MBA	Micromonosporales	*Micromonospora tulbaghiae* (jgi.1058868)	99.3
SH-108	R2A	Micromonosporales	*Micromonospora endophytica* (EU560726)	99.5
SH-115	SCAM	Corynebacteriales	*Rhodococcus nanhaiensis* (JN582175)	99.9

[a]: indicates the percent sequence similarity shared between each isolate and its nearest type strain.

Table S2. 16S rRNA taxonomic affiliation of 17 Bacillales (order) strains isolated from *Scopalina hapalia*.

Isolate	Isolation Medium	Family	Closest relative in EzBioCloud (Access Number)	Identity (%) [a]
SH-02a	A1BFe+c	Bacillaceae	*Bacillus paralicheniformis* (KY694465)	100.0
SH-02b	A1BFe+c	Bacillaceae	*Bacillus paralicheniformis* (KY694465)	100.0
SH-02c	A1BFe+c	Bacillaceae	*Bacillus paralicheniformis* (KY694465)	100.0
SH-04	A1BFe+c	Bacillaceae	*Bacillus licheniformis* (AE017333)	99.9
SH-10	LB	Bacillaceae	*Bacillus paralicheniformis* (KY694465)	99.9
SH-22	R2A	Bacillaceae	*Bacillus paralicheniformis* (KY694465)	100.0
SH-42	MYA2	Bacillaceae	*Bacillus paralicheniformis* (KY694465)	99.6
SH-46	SCAM	Bacillaceae	*Bacillus licheniformis* (AE017333)	99.9
SH-60	LB	Bacillaceae	*Bacillus paralicheniformis* (KY694465)	100.0
SH-68a	MYA2	Bacillaceae	*Bacillus licheniformis* (AE017333)	99.8
SH-68b	MYA2	Bacillaceae	*Bacillus licheniformis* (AE017333)	99.8
SH-99	MBA	Bacillaceae	*Bacillus licheniformis* (AE017333)	99.8
SH-100	MBA	Bacillaceae	*Bacillus licheniformis* (AE017333)	99.8
SH-116a	MBA	Bacillaceae	*Bacillus licheniformis* (AE017333)	99.8
SH-137	MBA	Bacillaceae	*Bacillus berkeleyi* (JN187498)	98.8
SH-32	MBA	Thermoactinomycetaceae	DQ448769_s	100.0
SH-39 [b]	MBA	Thermoactinomycetaceae	DQ448769_s	97.1

[a]: indicates the percent sequence similarity shared between each isolate and its nearest type strain, [b]: represents a potential new phylotype.

Table S3. Taxonomic affiliation of the three fungal isolates from *Scopalina hapalia* after sequencing fragments containing the Internal Transcribed Spacer (ITS) region, as well as partial beta-tubulin and calmodulin genes.

Isolate	Isolation Medium	Order	Closest Relative in the in-House Sequence Database of Westerdijk Fungal Biodiversity Institute
SH-53	A1BFe+c	Trichosphaeriales	*Nigrospora aurantiaca* Mei Wang & L. Cai
SH-122	A1BFe+c	Eurotiales	*Aspergillus sydowii* (Bainier & Sartory) Thom & Church
SH-123	LB	Sordariales	*Chaetomium globosum* Kunze
